# Functional echocardiography identifies association between early ventricular dysfunction and outcome in pediatric sepsis

**DOI:** 10.3389/fped.2025.1570519

**Published:** 2025-06-03

**Authors:** Sonia Reveco, Stella Barbagelata, Pablo Cruces, Franco Diaz, Karla Yohanessen, Marcos Larraín, Mario Guerra, Alexander Bataszew

**Affiliations:** ^1^ Pediatric Intensive Care Unit, Hospital El Carmen Dr Luis Valentin Ferrada, Santiago, Chile; ^2^ Pediatric Intensive Care Unit, Hospital San Juan de Dios, Santiago, Chile; ^3^ Pediatric Intensive Care Unit, Clínica Dávila, Santiago, Chile; ^4^ Facultad de Ciencias de la Vida, Universidad Andres Bello, Santiago, Chile; ^5^ Facultad de Medicina, Universidad Finis Terrae, Santiago, Chile; ^6^ Facultad de Medicina, Universidad de Chile, Santiago, Chile; ^7^ Pediatric Intensive Care Unit, Hospital Roberto del Río, Santiago, Chile

**Keywords:** sepsis, septic shock, echocardiography, children, hemodynamics, monitoring

## Abstract

**Objective:**

This feasibility study aimed to describe the relation between ventricular dysfunction and outcome in pediatric sepsis.

**Methods:**

This prospective observational multicenter study was conducted in two Pediatric Intensive Care Units (PICU). We enrolled 51 patients aged younger than 15 year-old diagnosed with sepsis or septic shock. Functional echocardiography was performed by a pediatric intensivist within the first 24 h of admission and blind validated by a pediatric cardiologist. Ventricular dysfunction was defined by the presence of left or right systolic and/or diastolic dysfunction. The absence of these findings was considered normal ventricular function. Outcome was assessed by septic shock diagnosis rate, pediatric adaptation of Sequential Organ Failure Assessment (pSOFA), cardiovascular component of pSOFA, PICU-free and ventilator-free days.

**Results:**

29 patients had sepsis, and 22 had septic shock. The main sites of infection were pulmonary (58.8%) and abdominal (17.6%). One out of four had ventricular dysfunction, and this group presented higher frequency of septic shock (69.2% vs. 34.2%, *p* = 0.028), higher frequency of total pSOFA ≥3 at 24 h (92% vs. 64%, *p* = 0.04), cardiovascular component of pSOFA (69.2% vs. 31.2%, *p* = 0.017), and fewer PICU-free days [18 [0–23] vs. 23 [18–25], *p* = 0.027], compared to normal ventricular function group. Additionally, there were more abnormal tissue doppler measurements, lower ś wave Z-Score [−0.6 [−1.3;0.4] vs. 0.5 [−0.2;1.1], *p* = 0.01] and lower é wave Z-Score [1.5 [−2;0,1] vs. −0.3 [−2;0.4], *p* = 0.03] in the ventricular dysfunction group.

**Conclusion:**

Ventricular dysfunction was associated with more sepsis severity at 24 hours, fewer PICU—free days. Tissue doppler parameters were related to ventricular dysfunction.

## 1 Introduction

Sepsis is a common condition in pediatrics (1). Every year, approximately 25 million children experience sepsis worldwide, resulting in more than 3 million deaths and physical, cognitive, emotional, and psychological sequelae with long-term effects on patients and their families, being an at-risk population, especially in limited resources settings (2).

There has been a strong interest in identifying phenotypes and endotypes of sepsis that reflect individual biology and may identify subgroups of patients more likely to benefit from specific therapeutic interventions (3). In pediatric sepsis, phenotyping is based on the trajectory of organ dysfunction and some biomarkers. However, its clinical translation is limited because it requires specific laboratory techniques that aren't widely available for decision-making during the acute phase of the disease (4).

Echocardiography has emerged as a crucial component in the evaluation of patients with sepsis, due to its non-invasive nature and wide availability moving from a cardiologist's detailed anatomical point of view to a systematized functional assessment by intensivists. Recent studies have shown that children with septic shock exhibit increased myocardial dysfunction compared to those without shock (5). Furthermore, the presence of left ventricular dysfunction has been associated with mortality in children and adults with septic shock (6, 7). Most of the studies face some criticism because of the interdependence of the echocardiographic measurements. Thus, there is a growing interest in assessing myocardial flow velocity by tissue Doppler imaging (TDI) because of its theoretical advantage of being independent of preload and afterload (8). We planned this study to prospectively evaluate ventricular dysfunction throughout functional echocardiography in children with sepsis and determine their association with outcomes. We hypothesize that there is an association between early ventricular dysfunction and worst outcomes. Also we hypothesize that tissue Doppler parameters could be related to early ventricular dysfunction in septic pediatric patients.

## 2 Materials and methods

Our study was conducted in two pediatric intensive care units (PICUs) in Santiago de Chile: Hospital El Carmen de Maipú (7-bed general PICU) and Hospital Roberto del Río (32-bed general PICU) between December 1st, 2022, and August 30th, 2023. This study was approved by their respective IRBs (ID 81/2023 and ID 046/2022), and written consent was obtained from legal guardians. Procedures were performed according to the ethical standards of institutional responsibility in human research and the 1975 Declaration of Helsinki.

We enrolled patients younger than 15 years-old who were admitted to the PICU for sepsis or septic shock. Sepsis and septic shock were defined according to the Pediatric Consensus Conference Criteria (9) as follows: sepsis was diagnosed when a patient presented with a suspected or confirmed infection and concomitant systemic inflammatory response syndrome; septic shock was diagnosed when a patient manifested cardiovascular dysfunction in the setting of sepsis. Cardiovascular dysfunction was defined as hypotension for age despite 40 ml/kg isotonic fluid resuscitation within 1 hour; need for vasoactive support (dopamine above 5 micrograms/kg/minute or dobutamine, epinephrine, and/or norepinephrine at any dose) to maintain an age-appropriate blood pressure (BP); and/or two of the following: (a) unexplained base deficit greater than 5 mEq/L, (b) arterial lactate greater than 2-times the upper limit of normal, (c) capillary refill greater than or equal to 5 s, (d) urine output less than 0.5 ml/kg/hour, (e) core-to-peripheral temperature gradient greater than or equal to 3 degrees Celsius. Patients with uncorrected congenital heart diseases, intracranial hypertension, major thoracic burns, extracorporeal membrane oxygenation support, and end-of-life care were excluded. Demographic variables, such as age, sex, weight, height, race, comorbidities, sites of infection, and etiology were recorded.

Functional echocardiographic was performed within 24 hours of PICU admission by two pediatric intensivists (SB and SR). Both physicians had at least 12 months of training in functional echocardiography. General Electric Vivid 7 and SonoSite Edge equipment were used depending on their availability. Image acquisition was done independently from the clinical team within the first 24 hours after admission. All images and assessment were subsequently blindly validated by an expert cardiologist with more than 5 years of experience. (ML and MG).

The following qualitative and quantitative functional parameters were assessed: Left ventricular (LV) systolic and diastolic function [including TDI], right ventricular (RV) systolic and diastolic function, and cardiac index.

a) LV systolic function
  a.1) Shortening fraction (SF) is defined as the percent change in LV dimension from end-diastole to end-systole based on either M-mode or 2D imaging acquired just below the level of the mitral valve leaflet tips.
    SF%=(LVEDD−LVESD)/LVEDD×100

LVEDD: left ventricular end-diastolic dimension.

LVESD: left ventricular end-systolic dimension

a.2) Ejection fraction (EF): defined as a percentage change in LV volume from end-diastole to end-systole.
  EF%=(LVEDV−LVESV)/LVEDV×100

LVED is the LV volume at end-diastole, and LVESV is the LV volume at end-systole.

a.3) Mitral annular plane systolic excursion (MAPSE): is a measurement of the mitral annular excursion during a cardiac cycle. It was standardized to the Z-score according to Koestenberger et al. (10)
a.4) Left ventricular tissue Doppler s’ (LV TDI s’) (cm/s): Assesses lateral mitral annulus velocity during different phases of the cardiac cycle. Pulsed tissue Doppler imaging was used to quantify myocardial muscle systolic velocity. It was standardized according to Eidem et al. These correspond to Z-Score values (11).

b) LV diastolic function
  b.1) Left ventricular Doppler e’ (LV TDI e’) (cm/s): Assesses lateral mitral annulus velocity during different phases of the cardiac cycle. Pulsed tissue Doppler imaging was used to quantify the diastolic velocity of the myocardial muscle. It was standardized according to Eidem et al. These correspond to Z-Score values (11).
  b.2) E/e': E is the maximum velocity of blood flow during rapid or passive transmitral filling, and E ´ is the maximum velocity away from the mitral annulus in early diastole; it is posited as early diastolic dysfunction with a cut-off point of 10.

c) RV function
  c.1) Tricuspid Annular Plane Systolic Excursion (TAPSE): is a measurement of the tricuspid annular excursion during a cardiac cycle, assessing systolic dysfunction. It was standardized to the Z-Score according to Koestenberger et al. (10)
  c.2) Tricuspid Pulsed Doppler: wave E/A, assessing diastolic dysfunction.

d) Cardiac index: cardiac output indexed by body surface area where applicable:

- Stroke Volume (SV) = left ventricular outlet tract (LVOT) area × time velocity integral (TVI): LVOT Area = (Aortic diameter/2) (2)

Cardiacoutput(CO)=Heartrate×SV=(L/min)

$$\mathrm{Cardiac}\;\mathrm{index}=\mathrm{CO}/\mathrm{Body}\;\mathrm{surface}\;\mathrm{area}=(L/\min/m^{2})$$

f) Inferior vena cava (IVC) collapsibility index: is calculated by the following formula:

IVC collapsibility index = [maximum diameter on expiration—(minimum diameter on inspiration/maximum diameter on expiration)]

f) IVC distensibility index = [(maximum diameter on inspiration–minimum diameter on expiration)/minimum diameter on expiration]

g) Qualitative: f.1) contractility biventricular; f.2) paradoxical movement of the interventricular septum; f.3) RV/LV ratio; f.4) mitral coaptation; and f.5) cavities size.

These measurements were conducted in accordance with the guidelines and standards for pediatric echocardiography established by the American Society of Echocardiography (12). Images not meeting high-quality standards per the cardiologist's assessment were excluded from the analysis.

Based on qualitative and quantitative echocardiographic parameters and the cardiologist's expertise, we classified patients as follows:

1) LV systolic dysfunction: defined as EF < 55% and/or MAPSE < −2 SD and/ or SF < 30%
2) LV diastolic dysfunction: E/A wave inversion by pulsed mitral Doppler, E/é ratio > 10.
3) RV dysfunction: defined as systolic assessment through TAPSE < −2 SD, diastolic dysfunction with E-Á wave inversion with tricuspid pulsed Doppler, qualitative signs of RV overload (right ventricular dilatation, paradoxical movement of the ventricular septum).
4) Hypovolemia: small cavities, mitral coaptation, IVC collapsibility index > 50%, and IVC distensibility index > 20% with normal or LV hyperdynamic function.
5) LV Hyperdynamic: There were no findings of hypovolemia. IVC with collapsibility < 30% and EF > 60%.
6) Well-resuscitated: Normal quantitative and qualitative parameters. Cardiac Index between 2.5–6l/min/m^2^.

When two or more findings were identified, the cardiologist carefully reviewed the images and selected the predominant one.

Based on the above echocardiographic findings, we classified patients in ventricular dysfunction group, corresponding to LV systolic dysfunction, LV diastolic dysfunction, RV dysfunction; and normal ventricular function corresponding to hypovolemia, LV hyperdynamic, and well-resuscitated.

Organ dysfunction was assessed using the pediatric Sequential Organ Failure Assessment (13) (pSOFA) at 24, 72, and 168 h after PICU admission. pSOFA score assesses six end-organ dysfunctions: respiratory, cardiovascular, coagulation, neurological, hepatic, and renal, with a score of 0–4 points each. Total pSOFA score is the sum of the worst score of each system during a 24-hour period, ranging between 0 and 24, with higher scores indicating worse outcomes (13). We used the cardiovascular component of pSOFA (CV-pSOFA) and a cut-off point of total pSOFA ≥ 3 or more based on the worst outcome identified in a previous study of pediatric sepsis (13). Ventilator-free and PICU-free days were assessed at 28 days after admission.

### 2.1 Statistical analysis

The results were expressed as proportions (%) or medians (interquartile range). We performed the chi^2^ test and Fisher's exact test to compare frequencies between groups and the Mann–Whitney *U*-test for comparisons of continuous variables. *p* < 0.05 was considered significant.

## 3 Results

The echocardiographic assessment for the study was done on 52 patients. After the cardiologist review, one case was excluded due to poor quality of images, with a 98% agreement of intensivists and cardiologists. Fifty-one patients were included in the analysis; 90% were Hispanic, and 47.5% of patients were female. The median age was 11.9 months (0.6–172.5). Clinical and demographic variables are shown in Table 1. Twenty-nine patients had the diagnosis of sepsis, and the remaining 22 had septic shock. The main sites of infection were pulmonary (58%), abdominal (17.6%), and central nervous systems (7.8%). The most common comorbidities were preterm birth, bronchopulmonary dysplasia, and genetic or neurological disorders (Table 1).

**Table 1:** Demographic clinical characteristics of patients according to ventricular dysfunction and minor echocardiographic involvement.

Characteristics clinical	All patients (*n* = 51)	Ventricular dysfunction (*n* = 13)	Normal ventricular function (*n* = 38)
Age (months)	12	9	13
Weight (kg)	9.8	8.8	10
Height (cm)	80	76	80
Female sex	24/51 (47%)	6/13 (46.1%)	18/38 (47.4%)
Race			
Hispanic	46/51 (90%)	13/13 (25%)	33/38 (65%)
Afro-caribbean	5/51 (10%)	–	5/38 (10%)
Site of infection			
Respiratory	30/51 (58.8%)	8/13 (61.5%)	22/38 (57.9%)
Abdominal	9/51 (17.6%)	2/13 (15.4%)	7/38 (18.4%)
Central nervous system	4/51 (7.8%)	2/13 (15.4%)	2/38 (5.3%)
Other	8/51 (15.6%)	1/13 (7.7%)	7/38 (18.4%)
Ethiology			
Viral	22/51 (43%)	6/13 (46.2%)	16/38 (42.1%)
Bacterial	21/51 (41.2%)	5/13 (38.5%)	17/38 (44.7%)
Comorbidities			
No comorbidities	35/51 (68.6%)	9/13 (69.2%)	36/38 (68.4%)
Neurological	5/51 (9.8%)	2/13 (15.4%)	3/38 (7.9%)
Preterm birth	7/51 (13.7%)	2/13 (15.4%)	5/38 (13.2%)

Age, weight, height are informed as median. The rest of the data are presented as number.

Both groups presented no differences in demographic characteristics. The total pSOFA score was ≥3 in 70% at 24 h, 56% at 72 h, and 15% at 168 h (Table 2).

**Table 2:** General outcomes.

Outcomes	All patients (*n* = 51)	Ventricular dysfunction (*n* = 13)	Normal ventricular function (*n* = 38)	*p*-value
PIM 3	1.4 (0.4; 3.3)	1.4 (0.8; 5.1)	1.3 (0.4; 2.9)	0.61
Total pSOFA ≥ 3 (24 h)	36/51 (70.6%)	12/13 (92.3%)	24/38 (63.2%)	0.04
Total pSOFA ≥ 3 (72 h)	29/51 (56.9%)	9/13 (69.2%)	20/38 (52.6%)	0.29
Total pSOFA ≥ 3 (168 h)	8/51 (15.7%)	3/13 (23.1%)	5/38 (13.2)	0.39
Shock	22/51 (43.1%)	9/13 (69.2%)	13/38 (34.2%)	0.02
pSOFA CV component (24 h)	21/51 (41.2%)	9/13 (69.2%)	12/38 (31.2%)	0.01
pSOFA CV component (72 h)	11/51 (21.5%)	3/13 (23.1%)	8/38 (21%)	0.07
pSOFA CV component (168 h)	2/51 (3.9%)	0/13 (0%)	2/38 (5.3%)	0.07
Ventilator - free days	25 (22; 26)	25 (20; 26)	25 (22; 28)	0.15
PICU- free days	22 (16; 24)	18 (0; 23)	23 (18; 25)	0.02

PIM 3, pediatric index of mortality 3; Total pSOFA, pediatric sequential organ failure assessment; CV-pSOFA: cardiovascular component of pSOFA; PICU, pediatric intensive care unit; Shock: SIRS + Cardiovascular dysfunction according to Pediatric Consensus Conference Criteria (9). PIM 3, Ventilator—free days, PICU—free days data are presented as median (interquartile range). pSOFA and CV-pSOFA CV are presented as number (percent).

**Figure 1:**
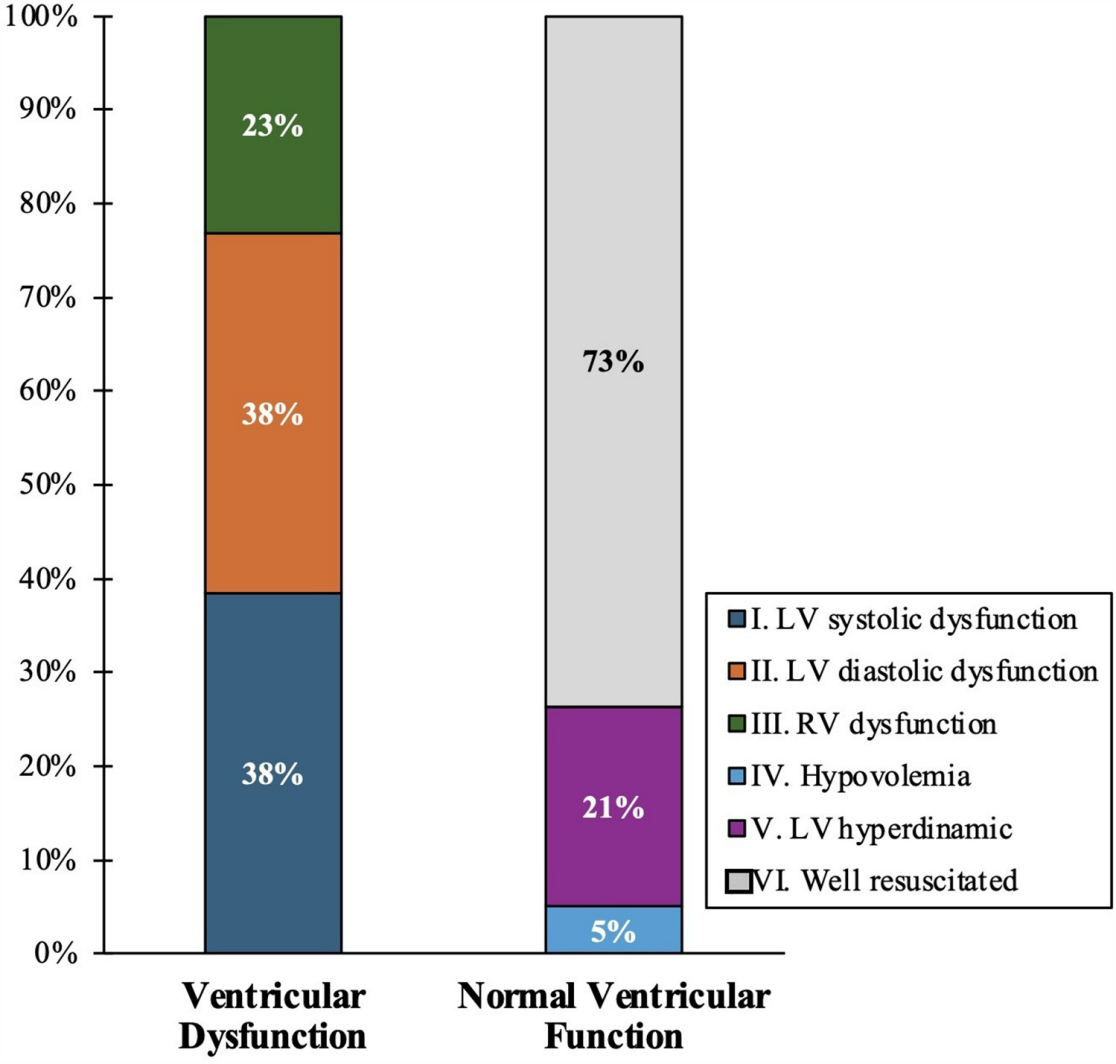
Echocardiographic groups according to ventricular dysfunction and normal ventricular function.

Regarding echocardiographic findings, the well-resuscitated one was the most common (54.9%). Ventricular dysfunction was identified in 25.5% of the patients, with normal ventricular function in the remaining 74.5% (Figure 1). Four patients (7.8%) presented with 2 findings, 2 of them corresponding to moderate to severe LV hyperdynamic and low LV diastolic dysfunction, which can be explained by alteration in the filling of the LV caused by the LV hyperdynamic. The other 2 cases presented LV systolic dysfunction and RV simultaneously.

Regarding quantitative echocardiographic parameters, the ventricular dysfunction group presented lower Z-Score LV TDI s’ [−0.6 (−1.3–0.44) vs. 0.5 (−0.2–1.1), *p* = 0.01], and more negative Z-Score LV TDI e’ [−1.5 (−2−−1) vs. −0.3 (−2–0.49, *p* = 0.027)]. There were no differences in the other quantitative parameters There were no patients defined as having systolic dysfunction solely by the śwave Z-Score. This means that although the śwave Z-Score was lower in the ventricular dysfunction group, they were still considered normal (Table 3).

**Table 3:** Quantitative echocardiographic variables.

Variable	Unit	All patients	Ventricular dysfunction	Normal ventricular function	*p*-value
		(*n* = 51)	(*n* = 13)	(*n* = 38)	
SF	%	38 (33; 45)	65 (58; 67)	38.5 (34; 47)	0.3
EF	%	70 (64; 77)	65 (55; 77)	70 (65; 76)	0.49
MAPSE	Z score	0.56 (−1.1; 1.31)	0.5(−1.4; 1.57)	1.5 (−0.9; 1.23)	0.83
TAPSE	Z score	0.33 (−2.5; 1.4)	0.33 (−2.5; 1.4)	0.49 (−2; 1.3)	0.87
E/é	ratio	7 (5.5; 9.2)	6.6 (5.1; 8.8)	8.8 (6.6; 5.5)	0.84
VTI	cm	15.2 (12.3; 18.65)	14.5 (12.3; 17)	16 (12.5; 20)	0.36
CI	(L/min/m^2^)	2.9 (2.2; 3.6)	2.9 (2.3; 4.33)	3.7 (2.8; 2.2)	0.46
LV TDI s’	Z score	0.44 (−0.6; 1.05)	−0.6 (−1.3; 0.4)	0.5 (−0.2; 1.1)	0.01
LV TDI é	Z score	0.64 (−0.29; −2)	1.5 (−2; 1.1)	0.3 (−2; 0.4)	0.02

SF, fractional shortening; EF, ejection fraction; TVI, velocity–time integral; LV TDI s`, left ventricular tissue Doppler ś; LV TDI é, left ventricular tissue Doppler é; MAPSE, mitral annular plane systolic excursion; TAPSE, tricuspid annular plane systolic excursion. Data are presented as median (interquartile range).

Ventricular dysfunction group had a higher frequency of septic shock (69.2% vs. 34.2%, *p* = 0.028), fewer PICU-free days [18 [0–23] vs. 23 [18–25], *p* = 0.027], higher frequency of total pSOFA score >3 or more at 24 h (92.3 vs. 63.2%, *p* = 0.046), and higher CV-pSOFA at 24 h (69.2 vs. 31.2%, *p* = 0.017) (Table 2). One patient with ventricular dysfunction died. All normal ventricular function patients survived.

## 4 Discussion

This prospective multicenter study examined functional echocardiographic findings in children with sepsis and septic shock. We found that ventricular dysfunction was associated with higher septic shock diagnosis rate, higher total pSOFA score, and CV-pSOFA, and fewer PICU-free days. Interestingly, in an exploratory analysis, we found that there were lower tissue doppler measurements in the ventricular dysfunction group, meaning that this could be a promising tool to establish early ventricular dysfunction in sepsis pediatric patients. More studies are required to confirm this hypothesis.

The association between ventricular dysfunction and worse outcomes has been previously reported in two studies on pediatric sepsis (14, 15) and in a meta-analysis of 9 pediatric studies with great heterogeneity (16, 17). We highlight our study's prospective and multicenter design and the inclusion of a blinded evaluation by an expert pediatric cardiologist, supporting the cardiac functional echocardiography and confirming the relevance of ventricular dysfunction in the outcome of pediatric sepsis.

The functional echocardiography protocol utilized in the study is based on a combination of quantitative and qualitative assessments designed to identify specific findings that predominate in children with sepsis or septic shock. Pediatric intensivists perform this functional echocardiography evaluation at bedside in accordance with usual training and recommendations. This protocol of functional echocardiography incorporates cardiac POCUS assessment, quantitative variables and tissue Doppler with the goal of optimizing the functional evaluation (18).

TDI is an ultrasound modality performed using pulsed Doppler with specialized filters to measure and visualize the velocity of myocardial tissue movement. Myocardial tissue generates flow displacements characterized by lower frequency and higher amplitude than blood flow signals. This technique enables the early detection of ventricular dysfunction, with the advantage of being independent of cardiac preload and afterload, thereby offering superior sensitivity compared to conventional echocardiographic methods. There are some limitations regarding the precise alignment with the myocardial ultrasound beam and sensitivity to myocardial motion (8)

We highlight the prospective nature of our research, with a unified and complete protocol with the inclusion of TDI, a measurement with significant results that associates LV TDI s’ and LV TDI e’ with ventricular dysfunction. Although a previous study showed results that differed from ours, we believe that the retrospective nature of their research and some methodological bias, like not including TDI in all patients, may account for the conflicting findings (16). Incorporating new technologies, like TDI, should be considered in future studies and at the bedside as an indicator of ventricular dysfunction in children with sepsis.

Of the 52 ultrasounds performed, only one was excluded, demonstrating a high level of concordance. Although previous studies have reported associations between MAPSE and TAPSE with mortality (15, 19) we did not find this in our research. We hypothesize that this discrepancy may be attributed to the higher proportion of patients with sepsis compared to septic shock in our cohort.

As a limitation of our study, we acknowledge the sample size and the echocardiographic assessment, which, even though performed early, consisted of a single measurement in time. We included IVC measurement in the hypovolemia finding, even though TVI measurement in ventilated patients controlled with 8 ml/kg suggests the best volume response (20). However, our patient cohort did not meet these conditions. Another limitation is that the various findings related to ventricular dysfunction were grouped into a single category. This prevents us from precisely determining whether a particular type of ventricular dysfunction predominantly contributes to adverse clinical outcomes. However, it is worth noting that a simple dichotomous classification is a practical approach, and it has been used in previous studies with large sample sizes (14). We added as a limitation from the statistical point of view the comparison of multiple results for each of these groups, which makes it exploratory since the correction for this was not made. It is important to note that in the exploratory analysis of TDI measurements, we did not find an association with outcomes. Although promising, the study was not powered to assess this hypothesis.

The strengths of our study include its conduction across two teaching centers, providing a representation of the Latin American context. Our protocol incorporated both qualitative and quantitative assessments, with echocardiograms performed by pediatric intensivists utilizing tissue Doppler imaging to complement both systolic and diastolic function. A cardiologist subsequently validated these assessments, underscoring the feasibility of implementing this tool at the bedside as a practical, regionally applicable approach.

In conclusion, identification of early ventricular dysfunction by functional echocardiography is associated with sepsis severity and fewer PICU-free days. A protocol of functional echocardiography might help to identify high-risk children and titration of individualized treatments early. The association of TDI alterations and ventricular dysfunction shows that TDI may be a promising tool to establish early ventricular dysfunction in the bedside assessment of children with sepsis that needs to be studied in the future.

## Data Availability

The original contributions presented in the study are included 1n the article/Supplementary Material, further inquiries can be directed to the corresponding author.
