# Supplementary figures and images for: Functional echocardiography identifies association between early ventricular dysfunction and outcome in pediatric sepsis
Source: Front Pediatr. 2025 Jun 3;13:1570519. doi: 10.3389/fped.2025.1570519 (PMC12170512; doi:10.3389/fped.2025.1570519)

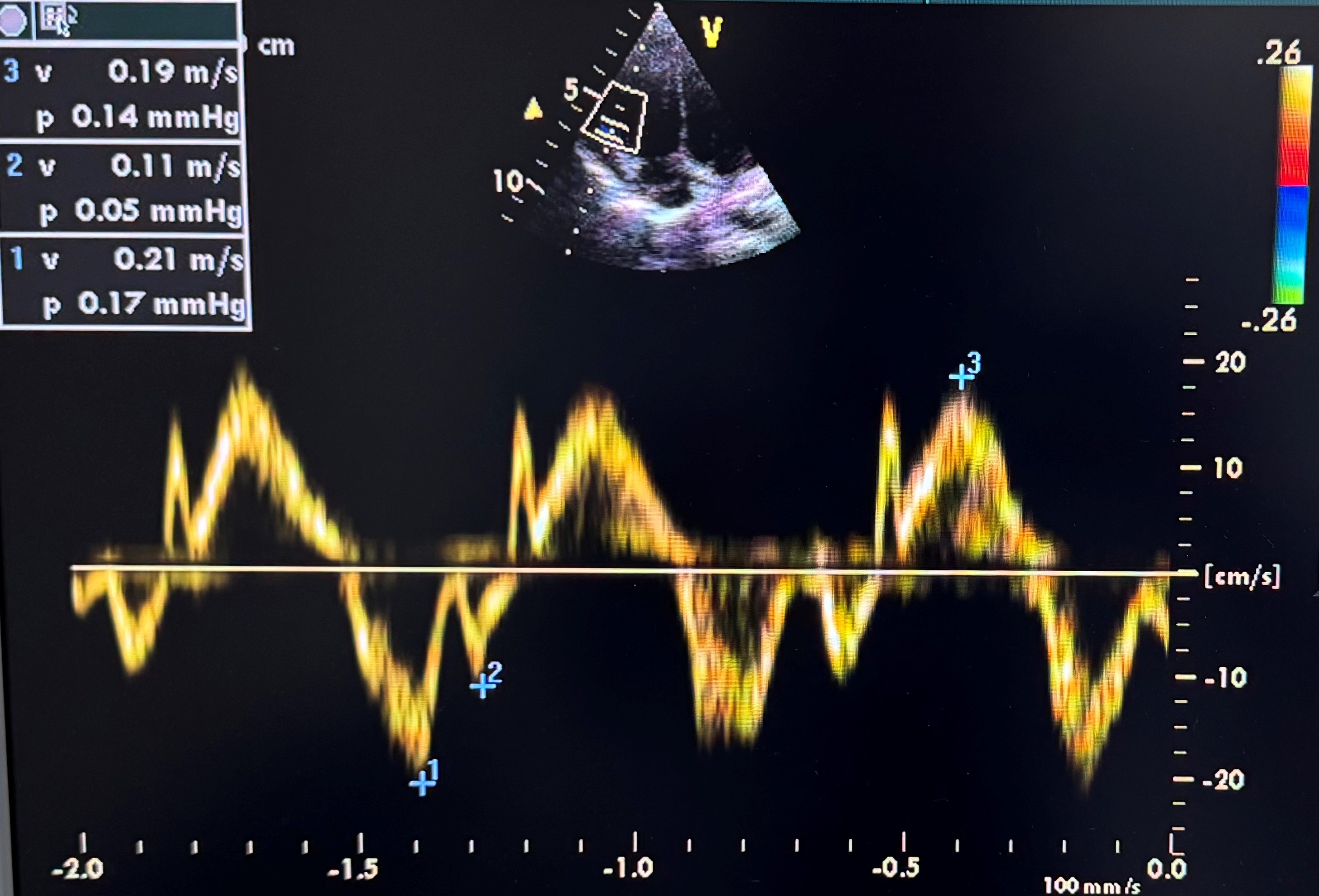

Supplement: Supplementary file 1 [file Image1.jpeg]

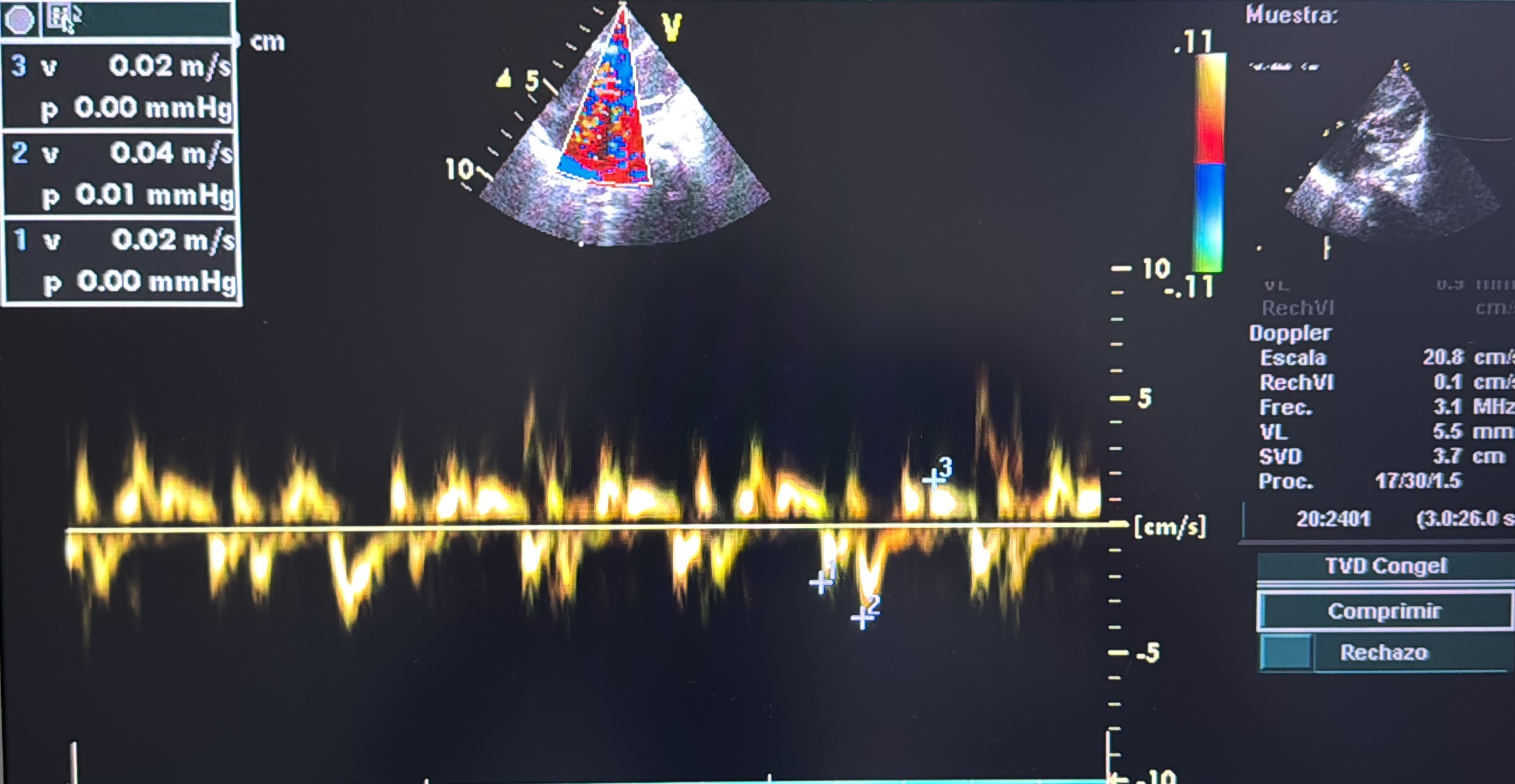

Supplement: Supplementary file 2 [file Image2.jpeg]
